# The First of Two One-Year, Multicenter, Open-Label, Repeat-Dose, Phase II Safety Studies of PrabotulinumtoxinA for the Treatment of Moderate to Severe Glabellar Lines in Adult Patients

**DOI:** 10.1093/asj/sjaa383

**Published:** 2021-05-04

**Authors:** Joely Kaufman-Janette, Rui L Avelar, Brian S Biesman, Zoe Diana Draelos, John E Gross, Derek H Jones, Mary P Lupo, Corey S Maas, Joel Schlessinger, Ava Teresa Shamban, Hema Sundaram, Susan H Weinkle, Vernon L Young

## Abstract

**Background:**

PrabotulinumtoxinA is a 900-kDa botulinum toxin type A produced by *Clostridium botulinum*.

**Objectives:**

The authors sought to investigate the safety of prabotulinumtoxinA for treatment of glabellar lines.

**Methods:**

This was a multicenter, open-label, repeat-dose, 1-year phase II safety study. Adults with moderate to severe glabellar lines at maximum frown, as assessed by the investigator on the validated 4-point photonumeric Glabellar Line Scale (0 = no lines, 1 = mild, 2 = moderate, 3 = severe), were enrolled. On day 0, patients received an initial treatment of 20 U prabotulinumtoxinA (4 U/0.1 mL freeze-dried formulation injected into 5 target glabellar sites). On and after day 90, patients received a repeat treatment (RT) if their Glabellar Line Scale score was ≥2 at maximum frown by investigator assessment. Safety was evaluated throughout the study.

**Results:**

The 352 study patients received a median total dose of 60 U, that is, 3 treatments per year. Fifty-one patients (14.5%) experienced adverse events (AEs) assessed as possibly study drug related; 11.1% experienced study drug-related AEs after the initial treatment. With each RT, progressively lower percentages of patients experienced study drug-related AEs. Six patients (1.7%) experienced study drug-related AEs of special interest: 3 eyelid ptosis (0.9%), 2 speech disorder (0.6%), and 1 blepharospasm (0.3%). Seven patients (2.0%) experienced serious AEs; none were study drug related. Of the 2393 samples tested, 2 patients (0.6%) tested positive for antibotulinum toxin antibodies at a single postbaseline visit.

**Conclusions:**

The safety of RTs of 20 U of prabotulinumtoxinA for moderate to severe glabellar lines was first established in this early phase II study based on a broad range of outcomes.

**Level of Evidence: 2:**

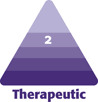

PrabotulinumtoxinA is a new 900-kDa botulinum toxin type A preparation produced by *Clostridium botulinum*. It was developed by Daewoong Pharmaceutical Co., Ltd. of Seoul, South Korea, and licensed to Evolus, Inc. of Newport Beach, CA (marketed in the United States under the trade name Jeuveau). Evidence that an early freeze-dried formulation of prabotulinumtoxinA was safe and effective for the treatment of moderate to severe glabellar lines in adult patients, and non-inferior to onabotulinumtoxinA (Botox Cosmetic, Allergan Inc., Irvine, CA), was first established in a 268-patient, randomized, double-blind, phase III comparator study conducted in South Korea.^1^ It was this early freeze-dried formulation that was also used in the first study initiated in the United States, which was the first of 2 US repeat-dose safety studies (EV-004). All subsequent studies conducted in the United States, including the second repeat-dose safety study (EV-006), were undertaken employing the final vacuum-dried commercial formulation. As with the final formulation, excipients included 0.5 mg human serum albumin and 0.9 mg NaCl/100 U vial. 

The EV-004 study was undertaken to investigate the safety of repeat treatments (RTs) of 20 U of prabotulinumtoxinA administered over the course of 1 year for moderate to severe glabellar lines in a large US adult population considered representative of the clinical population that typically might be seen for this condition. Safety endpoints examined were comprehensive and identical to those later utilized in the US pivotal, placebo-controlled, phase III EV-001 and EV-002 studies and in the second US repeat-dose study, EV-006.^2,3^ These included extent of exposure, total adverse events (AEs), common AEs, serious AEs, AEs of special interest (AESIs) as defined by the US Food and Drug Administration (FDA),^4^ study drug-related AEs, electrocardiogram and laboratory (hematology, chemistry, urinalysis, serum antibotulinum toxin antibodies) testing, vital signs, physical examination, and concomitant medications. All efficacy endpoints were considered exploratory.

## METHODS

### Study Design and Conduct

This was a multicenter, open-label (ie, non-blinded), non-randomized, long-term (ie, 1 year), repeat-dose study in which all patients received active treatment. It was primarily designed to collect long-term safety data related to repeat dosing of prabotulinumtoxinA in a representative patient population.

The EV-004 study was conducted between September 2014 and November 2015 at 11 study centers in the United States. The study protocol and its amendments were approved utilizing a centralized institutional review board review process by Quorum Review Institutional Review Board of Seattle, WA; all aspects of the study were conducted in accordance with the ethical principles originating from the 1975 Declaration of Helsinki and in compliance with the International Conference on Harmonisation harmonised tripartite guideline E6(R1): Good Clinical Practice. ClinicalTrials.gov identifier: NCT02184988.

### Patients

Study patients were selected from a population of healthy adults (≥18 years of age) with moderate (Glabellar Line Scale [GLS] score = 2) to severe (GLS score = 3) glabellar lines at maximum frown, as assessed by the investigator employing the validated 4-point photonumeric GLS (see Figure 1 of Beer et al^2^). Key exclusion criteria were previous treatment with botulinum toxin of any serotype in any area within the last 8 months or any planned treatment with botulinum toxin of any serotype during the study period; any previous facial aesthetic procedure in the glabellar area within the last 12 months; any other planned facial aesthetic procedure, or any surgery in the glabellar area, during the study; previous insertion of permanent material in the glabellar area; marked facial asymmetry; and presence or history of eyelid and/or eyebrow ptosis. Females of childbearing potential were required to have a negative pregnancy test and be willing to utilize an acceptable form of contraception. Prior to entering the study, all patients provided written informed consent.

**Figure 1.**
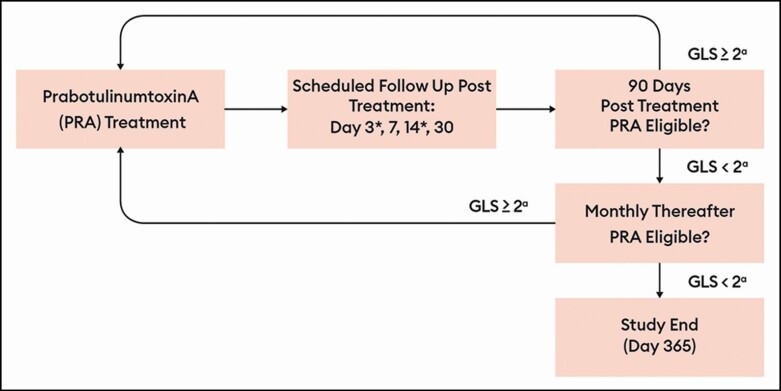
Treatment flowchart. Highlights of the study design included: (1) AE assessment at each visit. (2) Dose interval, ≥3 months. (3) Monthly visits after day 90 for ineligible patient to assess for repeat injection eligibility. (4) During repeat injection phase, day 3 and day 14 follow-up were conducted by phone and included a directed questionnaire. (5) No new treatment was offered after day 330. GLS, Glabellar Line Scale. ^a^At maximum frown by investigator assessment.

### Treatments and Follow-Up

On day 0, eligible patients received intramuscular injections of 20 U of prabotulinumtoxinA, administered as 4 U/0.1 mL injected into 5 target sites at least 1 cm above the bony orbital rim: the midline of the procerus, the inferomedial aspect of each corrugator muscle, and the superior middle aspect of each corrugator. Standardization of the treatment approach, total dose, and target injection sites is common to all registration studies for glabellar lines; in this setting, a clinician is not permitted the degree of latitude that he/she might otherwise exercise in their clinical practice. If required, topical anesthesia was allowed. After the initial treatment (IT) on day 0, patients were followed in the office on days 3, 7, 14, 30, and 90.

On and after day 90 (±7 days), patients were eligible for a RT if their GLS score was ≥2 at maximum frown, as judged by the investigator. Those patients who did not meet this criterion were followed monthly (±14 days) until eligible for RT or until the study ended on day 365. After a RT, patients were followed by telephone call from the investigator’s office on days 3 and 14; patients were followed by office visit on days 7, 30, and 90. Patients were to be followed for a maximum of 365 days from IT. No treatment was to take place after day 330 to ensure that there was at least 1 month of follow-up after the last injection. In total, eligible patients could have received up to 4 treatments (ie, the IT, and repeat treatments 1, 2 and 3 abbreviated as RT1, RT2, and RT3). A schematic of the RT evaluation cycle is presented in Figure 1.

### Assessments

In parallel with assessments carried out in the second repeat-dose EV-006 study, safety was evaluated by assessing the extent of exposure, AEs, medical histories, physical examination results, vital signs, electrocardiogram and laboratory (hematology, chemistry, urinalysis, and serum antibotulinum toxin antibodies) testing, and concomitant medications. Centralized facilities, independent of the sponsor, performed all laboratory and electrocardiogram testing. Hematology, chemistry, and urinalysis testing was performed at screening and end of study/early termination only. General botulinum toxin antibody testing was performed throughout the study at screening (before injection); IT days 30 and 90; each RT days 0 (before treatment), 30, and 90; and end of study/early termination. In the case where a patient tested negative for the presence of botulinum toxin antibodies at baseline, a positive result at a postbaseline visit would be indicative of seroconversion. In those select cases, specific testing for neutralizing antibodies—that is, a subset of antibodies that neutralize the activity of botulinum toxin, thus rendering it clinically ineffective—was also performed. Electrocardiogram testing was performed at screening, IT day 30 and end of study/early termination.

AEs were collected at each visit. To ensure that the reporting of AEs—particularly those of special interest—was comprehensive, a directed questionnaire and directed review of systems were employed to help guide the physical examination. Of note, the directed questionnaire was administered in person by the investigator or trained investigative site staff in a non-anonymous fashion during the site visit and recorded on paper in the patient’s source documents; the investigator alone was responsible for performing the subsequent directed review of systems and physical examination. AEs of special interest (AESIs), such as eyelid ptosis and speech disorder, were identified as those 50 AEs listed in the US FDA draft guidance document for developing botulinum toxin products for the treatment of upper facial lines.^4^

Efficacy outcomes were also evaluated at each clinic visit. These included investigator assessment on the GLS at maximum frown and at rest; patient assessment on a 5-point Global Aesthetic Improvement Scale (GAIS: 2 = much improved, 1 = improved, 0 = no change, −1 = worse, −2 = much worse); and patient assessment on a 5-point Subject Satisfaction Scale (SSS: 2 = very satisfied, 1 = satisfied, 0 = indifferent, −1 = unsatisfied, −2 = very unsatisfied).

### Outcomes and Statistical Analysis

Analyses were primarily descriptive in nature with continuous data summarized by number of patients, mean, standard deviation, median, minimum and maximum, and categorical data summarized by number and percentage of patients. Safety outcomes were reported for the safety population, which was defined as all patients who received at least 1 dose of prabotulinumtoxinA (ie, the IT on day 0). The Medical Dictionary for Regulatory Activities (MedDRA Version 17.0) was utilized to code and group AEs by system organ class and preferred term. AEs were summarized for each treatment—that is, following the IT, RT1, RT2, or RT3—as frequencies and proportions. The primary safety analysis was based on the proportion of patients with at least 1 AE that occurred from day 0 through day 365.

Exploratory efficacy outcomes were reported for the response-evaluable population, which was defined as all patients who received at least 1 dose of prabotulinumtoxinA on day 0 and had at least 1 postbaseline investigator or patient assessment. Only 1 efficacy analysis was conducted: the 95% CI was calculated for the proportion of patients with an improvement from day 0 of 1 point or more (ie, ≥1 point responders) on day 365 on the GLS at rest. Efficacy data were also summarized for various endpoints on each of days 3, 7, 14, 30, and 90 and at monthly follow-up visits thereafter. These endpoints included the proportion of patients with a ≥1-point improvement on the GLS at maximum frown, and the distributions of GAIS and SSS scores.

#### Sample Size

This was the first study initiated in the US prabotulinumtoxinA clinical development program. The sample size of approximately 350 enrolled patients was based on clinical judgment. Assuming a 15% drop-out rate, it was expected that 297 patients would complete the study. This number would allow for the observation of at least 1 AE with >95% probability if the incidence rate for that event was >0.85%.

## RESULTS

### Patient Disposition and Demographics

**Figure 2.**
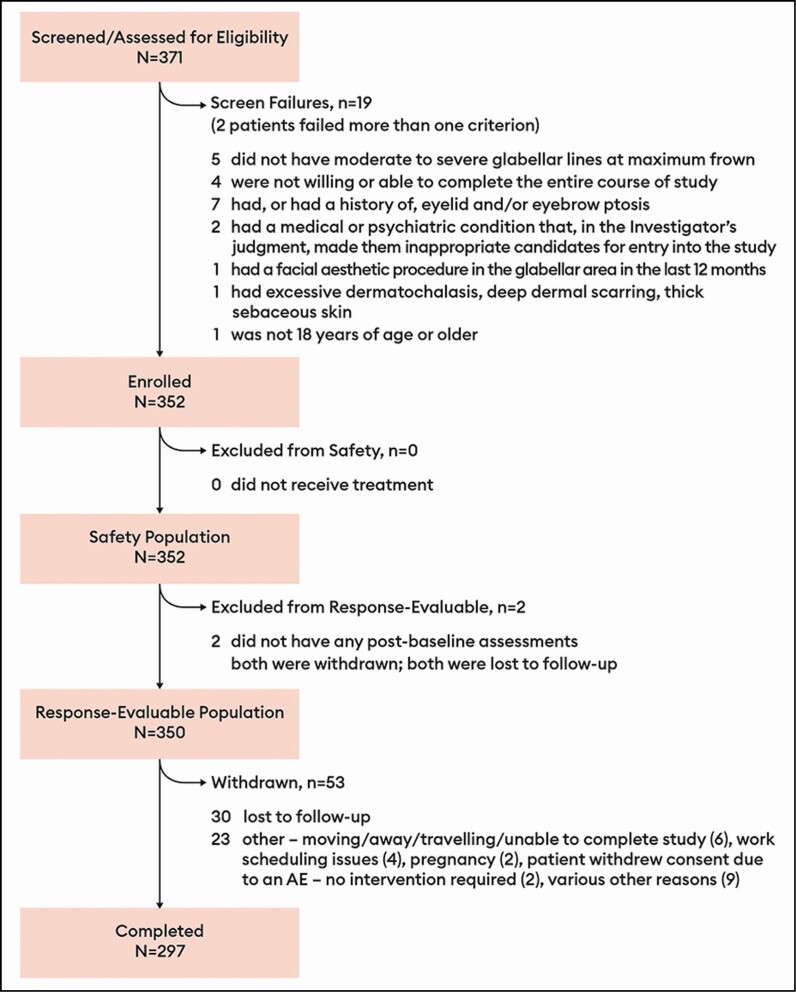
Disposition of all patients: safety and response-evaluable populations. The safety population was all patients who received at least 1 dose of prabotulinumtoxinA. The response-evaluable population was all patients who received at least 1 dose of prabotulinumtoxinA on day 0 and had at least 1 post-baseline investigator or patient assessment.

A total of 352 patients were enrolled, received at least the IT of 20 U prabotulinumtoxinA, and formed the safety population (Figure 2). All but 2 of these patients qualified for inclusion in the response-evaluable population. Most patients (297/352, 84.4%) completed the study; most commonly, patients who did not complete did not return and were lost to follow-up.

Patients had a mean age of 50.8 years (range of 23-83 years) (Table 1). Most patients (90.6%) were younger than 65 years; 9.4% (33/352) were 65 years or older. Most patients (94.0%) were female (331 vs 21 males). Most patients were racially identified as White (91.8%); 19.3% (68/352) were of Hispanic or Latino ethnicity. Of the 6 Fitzpatrick skin types, the most common were types II and III; 63.6% of patients were identified with these skin types. By investigator assessment, 71.3% of patients had severe glabellar lines at maximum frown at baseline (GLS score = 3). By investigator assessment, 92.3% of patients (n = 325) also had evidence of glabellar lines at rest (baseline GLS score >0).

**Table 1.** Demographic and Glabellar Line Characteristics at Baseline: Safety Population

Characteristic	PrabotulinumtoxinA (N = 352)	
Age (y)		
Mean ± SD [min, max]	50.8 ± 10.89 [23, 83]	
<65, n (%)	319	(90.6)
≥65, n (%)	33	(9.4)
Sex, n (%)		
Male	21	(6.0)
Female	331	(94.0)
Race, n (%)		
White	323	(91.8)
Black or African American	15	(4.3)
Asian	4	(1.1)
Other^a^	8	(2.3)
Multiple	2	(0.6)
Ethnicity, n (%)		
Hispanic or Latino	68	(19.3)
Not Hispanic or Latino	284	(80.7)
Fitzpatrick Skin Type,^b^ n (%)		
I	36	(10.2)
II	113	(32.1)
III	111	(31.5)
IV	77	(21.9)
V	12	(3.4)
VI	3	(0.9)
Investigator assessment of glabellar lines on the GLS, n (%)		
At maximum frown		
Moderate	101	(28.7)
Severe	251	(71.3)
At rest		
None	27	(7.7)
Mild	121	(34.4)
Moderate	148	(42.0)
Severe	56	(15.9)

GLS, Glabellar Line Scale; SD, standard deviation. ^a^All but 1 patient in the category of “other” identified as Hispanic or Latino. ^b^Type I = always burns, never tans (pale white skin); Type II = usually burns, tans minimally (white skin); Type III = sometimes burns, tans uniformly (cream/light brown skin); Type IV = rarely burns, always tans well (moderate brown skin); Type V = very rarely burns, tans very easily (dark brown skin); Type VI = never burns, deeply pigmented (dark brown to black skin).

### Safety

#### Extent of Exposure

The 352 patients in the safety population received a mean total dose of 61.8 U of prabotulinumtoxinA (range of 20-80 U) over the 1-year course of study; the median total dose was 60 U (3 treatments) (Table 2). Of the 297 study completers, 5 patients (1.7%) completed the study without requiring a RT; at no visit on day 90 or monthly thereafter were these patients assessed by the investigator to have a GLS score at maximum frown of 2 = moderate or 3 = severe. A further 43 patients (14.5%) received a single RT (mean of 206.7 days after the IT; range of 95-330 days), 98 patients (33.0%) received 2 RTs (means of 129.7 and 143.8 days after the initial and first RTs, respectively; ranges of 89-238 days and 84-233 days, respectively), and 151 (50.8%) received 3 RTs (means of 94.5, 98.3, and 99.8 days after the initial, first RT, and second RTs, respectively; ranges of 83-128 days, 77-156 days and 79-167 days, respectively) (Tables 2 and 3).

**Table 2.** Extent of Exposure, Summarized by Total Units of PrabotulinumtoxinA Injected and Total Number of Treatments Administered: Safety Population

Total drug administered	Study completers (N = 297)		All patients (N = 352)	
Total dose injected (U), mean ± SD [min, max]	66.6 ± 15.67 [20, 80]		61.8 ± 19.69 [20, 80]	
Median	80		60	
Total treatments administered, n (%)				
1 Treatment (IT only)	5	(1.7)	33	(9.4)
2 Treatments (IT + RT1)	43	(14.5)	57	(16.2)
3 Treatments (IT + RT1 + RT2)	98	(33.0)	108	(30.7)
4 Treatments (IT + RT1 + RT2 + RT3)	151	(50.8)	154	(43.8)
Dose interrupted, n (%)	1	(0.3)	1	(0.3)

IT, initial treatment; RT, repeat treatment.

**Table 3.** Extent of Exposure, Summarized by Number of Days Between PrabotulinumtoxinA Treatments: Study Completers Only (N = 297)

Total number of days between treatments	Mean ± SD [min, max]	Median
Patients who received only 1 treatment (n = 5)		
From IT to end of study	363.8 ± 3.63 [359, 369]	363.0
Patients who received a total of 2 treatments (n = 43)		
Between IT and RT1	206.7 ± 50.83 [95, 330]	202.0
From RT1 to end of study	161.4 ± 53.34 [28, 272]	172.0
Patients who received a total of 3 treatments (n = 98)		
Between IT and RT1	129.7 ± 28.75 [89, 238]	123.0
Between RT1 and RT2	143.8 ± 31.22 [84, 233]	139.5
From RT2 to end of study	94.2 ± 38.83 [28, 169]	95.0
Patients who received a total of 4 treatments (n = 151)		
Between IT and RT1	94.5 ± 10.03 [83, 128]	91.0
Between RT1and RT2	98.3 ± 18.04 [77, 156]	91.0
Between RT2 and RT3	99.8 ± 15.71 [79, 167]	92.0
From RT3 to end of study	73.3 ± 26.75 [21, 127]	83.0

IT, initial treatment; RT, repeat treatment.

#### Adverse Events

A total 148 patients (148/352, 42.0%) experienced a total of 265 AEs over the course of study (Table 4). Approximately 30% of all patients (104/352) experienced an AE following the IT, representing 70.3% of all patients (104/148) who experienced an AE at any time during this study. Progressively lower percentages of patients experienced AEs following each RT: 15.4% after RT1, 12.6% after RT2, and 10.4% after RT3. Similar trends were observed for AEs assessed by the investigator as study drug related, serious AEs, and AESIs (Tables 4-5). Note that, overall, few patients experienced these latter types of events, with no patients experiencing a study drug–related or serious AS following RT3, and no patients experiencing an AESI following either RT2 or RT3.

**Table 4.** Summary of Treatment-Emergent AEs: Safety Population

AE parameter	PrabotulinumtoxinA (N = 352)		
	n/N	(%)	Events, No.
AE parameter	PrabotulinumtoxinA (N = 352)		
	n/N	(%)	Events, No.
All AEs	148/352	(42.0)	265
Last treatment before onset^a^			
IT	104/352	(29.5)	148
RT1	49/319	(15.4)	63
RT2	33/262	(12.6)	37
RT3	16/154	(10.4)	17
Any serious AE	7/352	(2.0)	9
Last treatment before onset^a^			
IT	3/352	(0.9)	4
RT1	3/319	(0.9)	3
RT2	2/262	(0.8)	2
RT3	0/154	(0.0)	0
Any study drug–related AE	51/352	(14.5)	59
Last treatment before onset^a^			
IT	39/352	(11.1)	43
RT1	11/319	(3.4)	12
RT2	4/262	(1.5)	4
RT3	0/154	(0.0)	0
Any AE leading to study discontinuation	2/352	(0.6)	2
Any AE leading to death	0/352	(0.0)	0
Relationship to study drug			
Not related	97/352	(27.6)	206
Possibly related	37/352	(10.5)	44
Probably related	10/352	(2.8)	11
Definitely related	4/352	(1.1)	4
Severity			
Mild	80/352	(22.7)	159
Moderate	59/352	(16.8)	95
Severe	9/352	(2.6)	11
Frequency			
≥5%	54/352	(15.3)	65
Nervous system disorder, headache^b^	54/352	(15.3)	65

AE, adverse event; IT, initial treatment; n, the number of patients at each level of summarization; RT, repeat treatment. ^a^Percentages are based on the number of patients who received these treatments. ^b^System organ class and preferred term. A patient was counted once in the system organ class if they reported 1 or more events.

**Table 5.** Summary of Treatment-Emergent AESIs: Safety Population

AE parameter	PrabotulinumtoxinA (N = 352)								
	All			Study drug related			Not study drug related		
	n/N	(%)	Events	n/N	(%)	Events	n/N	(%)	Events
Any AESI	11/352	(3.1)	11	6/352	(1.7)	6	5/352	(1.4)	5
Last treatment before onset^a^									
IT	8/352	(2.3)	8	5/352	(1.4)	5	3/352	(0.9)	3
RT1	3/319	(0.9)	3	1/319	(0.3)	1	2/319	(0.6)	2
RT2	0/262	(0.0)	0	0/262	(0.0)	0	0/262	(0.0)	0
RT3	0/154	(0.0)	0	0/154	(0.0)	0	0/154	(0.0)	0
Onset, days since last treatment									
No. of events	11			6			5		
Mean ± SD	22.5 ± 34.96			8.0 ± 4.38			39.8 ± 48.40		
Median	10.0			9.0			11.0		
Minimum, maximum	1, 113			1, 13			3, 113		
Duration, d									
No. of events	11			6			5		
Mean ± SD	18.6 ± 17.63			20.3 ± 20.12			16.6 ± 16.18		
Median	18.0			19.5			18.0		
Minimum, maximum	1, 55			1, 55			2, 42		

AESIs were those 50 events potentially suggestive of distant spread of botulinum toxin effects identified in “Guidance for Industry. Upper Facial Lines: Developing Botulinum Toxin Drug Products.”^4^ One patient had 2 AESIs: 1 was study drug related and 1 was not. AE, adverse event; AESI, adverse event of special interest; IT, initial treatment; n, the number of patients at each level of summarization; RT, repeat treatment. ^a^Percentages are based on the number of patients receiving each treatment.

No deaths were reported. Two patients experienced AEs that led to study discontinuation (Table 4). Of these, 1 patient was reported to have experienced mild postprocedural worsening of another wrinkle above 1 eyebrow at rest with an onset 9 days after the IT; although not apparent from a review of the patient’s photographic record, this type of rare event has been known to occur in some patients as an involuntary overcompensatory frontalis response to paralysis of the glabellar lines. The other patient experienced mild headache the day of the IT. Both events that led to study discontinuation resolved, and both were assessed as probably related to treatment. Neither was assessed as serious. Most AEs (254/265, 95.8%) were mild or moderate in severity (Table 4). Nine patients experienced 11 events (11/265, 4.2%) that were severe. These included 2 headache, 2 reports of dysfunctional uterine bleeding in 1 patient, and 1 each of viral gastroenteritis, failure of a pacemaker/defibrillator, pancreatitis, basal cell carcinoma, breast cancer, malignant anorectal neoplasm, and endometrial hyperplasia. Only 1 severe event of headache with an onset the day of the RT1 visit was assessed as possibly study drug related; all other severe events were assessed as unrelated.

Seven patients (2.0%) experienced a total of 9 treatment-emergent AEs assessed by the investigator as serious (Table 4): 2 patients with basal cell carcinoma, 1 patient with both breast cancer and pancreatitis, 1 patient with 2 reports of dysfunctional uterine bleeding, and 1 patient each with malignant anorectal neoplasm, ovarian adenoma, and device failure of a pacemaker/defibrillator. No serious event was assessed as study drug related, and no one discontinued the study for this reason.

Fifty-one patients (14.5%) experienced a total of 59 AEs assessed by the investigator as study drug related (Table 4). Most of the 265 AEs (206/265, 77.7%) reported during the study were assessed as not related to study drug. Altogether, 4 events (1.5%) were assessed as definitely related, 11 (4.2%) as probably related, and 44 (16.6%) as possibly related. Headache was the event most commonly assessed as study drug related; 33 patients (33/352, 9.3%) experienced a headache assessed as either possibly (n = 26) or probably (n = 7) study drug related. None were assessed as definitely related.

Headache, reported by 15.3% of all patients, was also the most common AE (Table 4). It was the only event reported in 5% or more of patients. By preferred term, a total of 11 other types of AEs occurred in 1% or more of patients (in 4 or more patients). These included sinusitis (3.4%), influenza (2.6%), urinary tract infection (2.6%), bronchitis (2.3%), gastroenteritis viral (1.4%), eyelid ptosis (1.4%; see AESI below), nasopharyngitis (1.1%), upper respiratory tract infection (1.1%), hypertension (1.1%), injection site bruising (1.1%), and injection site pain (1.1%).

Eleven patients (3.1%) experienced AESIs, many of which were assessed as unrelated to study drug (Tables 5 and 6). Two AESIs were moderate in severity; all others were mild in severity. None was assessed as serious, and no patient discontinued the study due to one of these types of events. Six patients (1.7%) experienced a total of 6 AESIs that were assessed as possibly, probably, or definitely related to study drug (Tables 5 and 6). Of the 6 events, 4 were categorized as eye disorders and 2 were categorized as nervous system disorders. These events included 3 reports of eyelid ptosis (0.9%), 1 of blepharospasm (0.3%), and 2 of speech disorder (0.6%) (Table 6). Between 0.3% and 1.4% of patients experienced a study drug–related AESI following any given treatment (Table 5). The median time to onset of study drug–related AESIs was 9 days after the patient’s most recent treatment date, and the median duration was 19.5 days; all resolved. Of particular interest, the 3 study drug–related eyelid ptosis events (0.9%), with onsets of 7, 10, and 12 days after the IT, resolved within 55, 26, and 24 days of onset, respectively. All 3 patients received 1 or more additional treatments of prabotulinumtoxinA; none experienced a repeat ptosis event.

**Table 6.** Treatment-Emergent AESIs by System Organ Class, Preferred Term, Relatedness, Patient Number, and Severity: Safety Population

System organ class and preferred term, relationship to study drug (patient no., severity)	PrabotulinumtoxinA (N = 352)		
	n	(%)	Events
All AESIs	11	(3.1)	11
Eye disorders	7	(2.0)	7
Blepharospasm	1	(0.3)	1
Possibly related (410024, mild)	1	(0.3)	1
Eyebrow/eyelid ptosis	5	(1.4)	5
Eyelid	5	(1.4)	5
Not related (406013, mild; 410010, mild)	2	(0.6)	2
Possibly related (402002, moderate; 402013, moderate)	2	(0.6)	2
Probably related	0	(0.0)	0
Definitely related (403003, mild)	1	(0.3)	1
Presbyopia	1	(0.3)	1
Not related (403026, mild)	1	(0.3)	1
Respiratory, thoracic, and mediastinal disorders	2	(0.6)	2
Dyspnea	2	(0.6)	2
Not related (403014, mild; 406020, mild)	2	(0.6)	2
Nervous system disorders	2	(0.6)	2
Speech Disorder	2	(0.6)	2
Possibly related (410002, mild; 410019, mild)	2	(0.6)	2

At each level of summarization, a patient was counted once if the patient reported 1 or more events; however, a single patient may be represented at more than 1 level of summarization. AEs were coded using MedDRA Version 17.0. AESIs were those 50 events potentially suggestive of distant spread of botulinum toxin effects, identified in “Guidance for industry. Upper facial lines: developing botulinum toxin drug products.”^4^ AESI, adverse event of special interest; n, number of patients at each level of summarization.

Of the 33 patients (33/352, 9.4%) who were 65 years of age or older, 16 patients (16/33, 48.5%) experienced AEs. One of the 9 serious events (basal cell carcinoma) and 2 of the 6 study drug–related AESIs (1 mild blepharospasm, 1 mild speech disorder) that occurred during the study were reported in patients 65 years of age or older.

#### Laboratory Assessments, Vital Signs, and Electrocardiography Assessments

None of the changes from baseline values for any of the hematology, chemistry or urinalysis measures was particularly noteworthy. A total of 2393 serum samples were collected throughout the study and tested for the presence of antibotulinum toxin antibodies. No patients tested positive at the screening visit for the presence of botulinum toxin antibodies. Two patients (0.6%) showed evidence of seroconversion at a postbaseline visit, suggesting that they had developed anti-botulinum toxin antibodies after exposure to prabotulinumtoxinA. Of these, 1 patient tested positive at the end of study visit only (approximately 5.5 months after her last treatment, RT1). She was responsive to treatment at that visit, with a GLS score at maximum frown of 1, suggesting that the antibody did not neutralize the activity of the botulinum toxin. She had otherwise tested negative at all other visits during which the test was performed (screening, IT day 30, RT1 day 0, and RT1 day 30). The second patient tested positive at the RT1 day 30 visit only; she remained responsive to treatment throughout the study, completing the study with a GLS score at maximum frown of 1, again suggesting that the antibody did not neutralize the activity of the botulinum toxin. She also tested negative at all other visits during which the test was performed (screening, IT day 30, RT1 day 0, and end of study/early termination). In both cases, neutralizing antibody testing was also performed and was found to be negative, consistent with the clinical results that indicated the toxin remained effective.

None of the individual differences in the changes from baseline values were particularly noteworthy for any of the vital sign measures assessed. As summarized by the independent centralized electrocardiography facility, none of the electrocardiography findings observed were of concern for the overall cardiac safety of prabotulinumtoxinA.

### Efficacy

**Figure 3.**
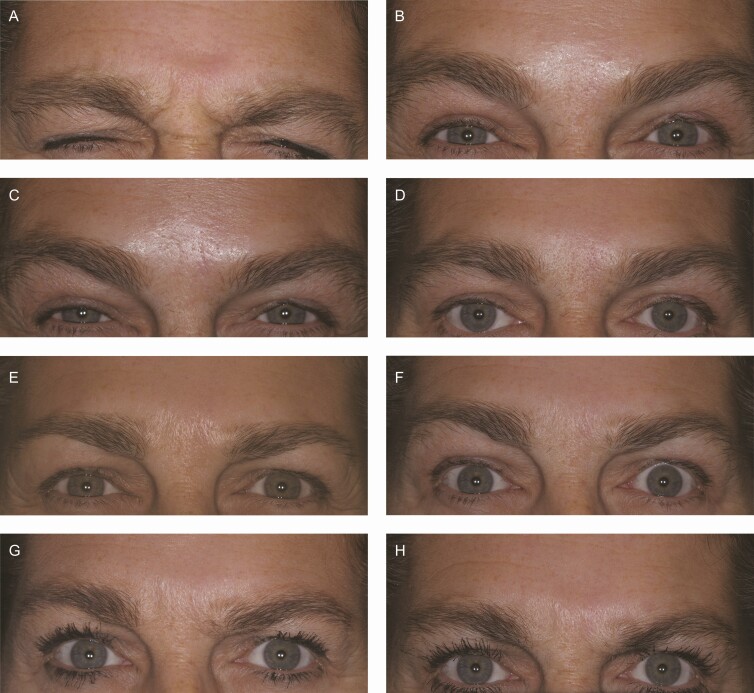
Photographs of glabellar lines at maximum frown at each of baseline (A) day 7 (B) day 14 (C) day 30 (D) day 90 (E) day 120 (F) day 150 (G) and day 180 (H) following initial treatment with 20 U prabotulinumtoxinA. This is a 52-year-old White female representative patient with Fitzpatrick skin type III and moderate glabellar lines at maximum frown at baseline. She received a single retreatment at 6 months (ie, at day 180) post-baseline.

Representative photographs of a patient’s glabellar lines at maximum frown taken at baseline and at days 7, 14, 30, 90, 120, 150, and 180 are presented in Figure 3A-H.

The proportion of patients in the response-evaluable population with a ≥1-point improvement from baseline GLS score at rest on day 365 was the only efficacy endpoint for which 95% CIs were constructed. Patients who qualified for this analysis (291 by investigator assessment) were limited to those who completed the study who also had evidence of glabellar lines at rest at baseline (a baseline GLS score at rest of >0). Of these, 75.9% of patients had a ≥1-point improvement from baseline GLS score at rest on day 365 by investigator assessment (95% CI 70.6, 80.7).

**Figure 4.**
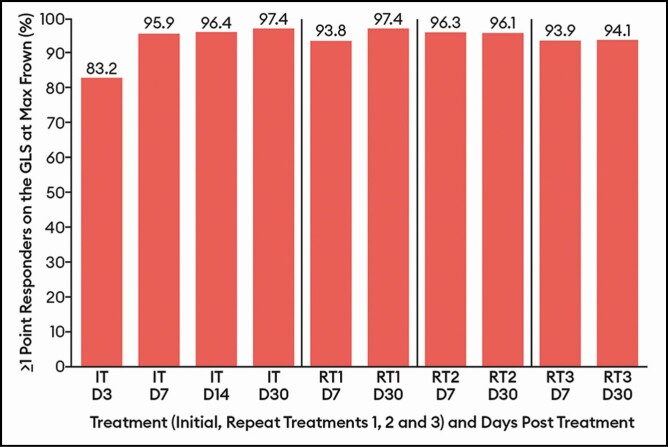
Percentage of patients with a decrease from baseline of ≥1 point on the Glabellar Line Scale at maximum frown by treatment at select visits by investigator assessment: response-evaluable population. Efficacy assessments were not performed at repeat treatments D3 or D14. On and after day 90, patients were eligible for a repeat treatment if their Glabellar Line Scale score was ≥2 at maximum frown, as judged by the investigator. If a patient did not have a Glabellar Line Scale score ≥2, they were followed monthly until eligible for repeat treatment, or until the study ended on day 365. D, day; IT, initial treatment; RT, repeat treatment.

**Figure 5.**
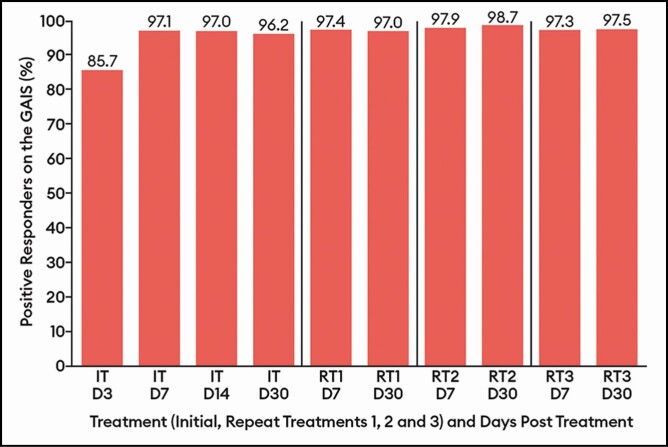
Percentage of patients with a positive response (improved/much improved) on the Global Aesthetic Improvement Scale by treatment at select visits by patient assessment: response-evaluable population. Efficacy assessments were not performed at repeat treatments D3 or D14. D, day; IT, initial treatment; RT, repeat treatment.

**Figure 6.**
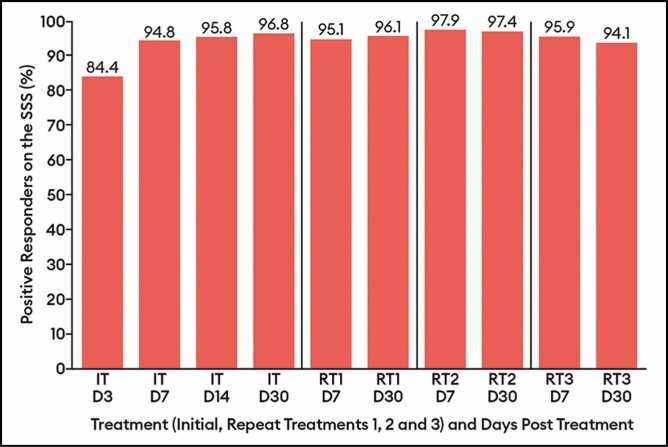
Percentage of patients with a positive response (satisfied/very satisfied) on the Subject Satisfaction Scale by treatment at select visits: response-evaluable population. Efficacy assessments were not performed at repeat treatments D3 or D14. D, day; IT, initial treatment; RT, repeat treatment.

A marked response to treatment was evident from the first assessment day (day 3) following the IT (Figures 4-6); on that day, 83.2% of patients had achieved a ≥1-point improvement on the GLS at maximum frown. As illustrated in Figure 4, the percentage of patients with a ≥1-point improvement on the GLS at maximum frown peaked from the day 7 to day 30 visits for each treatment. The percentages of patients with these outcomes at similar time intervals did not vary widely across RTs. For example, by investigator assessment and compared with 97.4% of patients at IT day 30, 97.4% at RT1 day 30, 96.1% at RT2 day 30, and 94.1% at RT3 day 30 experienced a ≥1-point improvement on the GLS at maximum frown (<4% absolute difference across treatments). A similar observation was noted for the percentage of patients with a ≥2-point improvement from baseline—that is, by investigator assessment and compared with 83.3% of patients at IT day 30, 85.2% at RT1 day 30, 86.1% at RT2 day 30, and 81.5% at RT3 day 30 experienced a ≥2-point improvement on the GLS at maximum frown (a <5% absolute difference across treatments; data not displayed).

The percentage of patients with a positive response ( improved/much improved) on the GAIS showed little variation across treatments, ranging between 96.2% and 98.7% at the day 7 and day 30 visits for all treatments (Figure 5). Similarly, the percentage of patients with a positive response (satisfied/very satisfied) on the SSS did not vary widely across treatments, ranging between 94.1% and 97.9% at the day 7 and day 30 visits for all treatments (Figure 6).

## DISCUSSION

The design and objectives of the EV-004 study closely resembled those of the later EV-006 study.^3^ Both were 1-year, open-label, phase II studies designed to investigate the safety of repeat doses of 20 U prabotulinumtoxinA for the treatment of glabellar lines. Where they differed was in the number of patients studied (N = 352 in EV-004; N = 570 in EV-006), in the scheduling of the first posttreatment visit (day 3 in EV-004; day 2 in EV-006), in the GLS assessments (by investigator only in EV-004; by both investigator and patient in EV-006), in the GAIS assessments (by patient only in EV-004; by both investigator and patient in EV-006), and in the product formulation process employed (lyophilized/freeze-dried in EV-004; vacuum-dried in EV-006). Of these, it is this latter difference (expanded on in the paragraph below) that is thought to have given rise to the 2 cases of seroconversion noted in the EV-004 study. Of the 2393 samples tested, 2 patients tested positive for the presence of botulinum toxin antibodies after exposure to prabotulinumtoxinA at a postbaseline visit. Importantly, no evidence of neutralizing antibodies that would have rendered the toxin clinically ineffective was found, and both patients remained responsive to treatment. No cases of seroconversion were observed in the EV-006 study or in either of the 2 placebo-controlled phase III studies of the US clinical development program (EV-001, EV-002), all of which utilized the final, vacuum-dried commercial formulation.^2,3^ No other impact on safety outcomes was evident.

Two different processes were employed to remove water from the vial in the final formulation of product for the prabotulinumtoxinA studies. The earlier freeze-drying technique, which was utilized in our study, required a greater overage of the active ingredient to yield 100 U of activity in the end product. It is thought that the formation of ice crystals during this process led to the disruption of the fragile protein structure, resulting in more inactive protein and a higher protein load within the final drug product, which then resulted in an increased probability of an immunological reaction. This theory is consistent with results reported by Jankovic et al^5^ in 2003 in which the higher protein load of the original formulation (25 ng protein/100 U) of onabotulinumtoxinA proved to be a risk factor for the development of blocking antibodies and immunoresistance, a risk that was mitigated by a subsequent formulation containing markedly less neurotoxin complex protein (5 ng protein/100 U). In contrast to the freeze-dried formulation utilized for our EV-004 study, the latter commercial vacuum-dried formulation proved to be a gentler processing technique on the botulinum protein complex, requiring little to no overage of this active ingredient to yield the same result. Importantly, no seroconversions were observed among any of 1062 prabotulinumtoxinA-treated patients who received the vacuum-dried formulation through their participation in the 2 single-dose phase III studies (EV-001, EV-002) and the other long-term, repeat-dose study (EV-006),^2,3^ supporting the theory that significantly decreasing the protein load minimizes the risk of antibody formation. In the end, both formulations achieved the same potency (the same amount of active botulinum toxin), as standardized by the LD-50 assay and as evidenced by comparing effectiveness outcomes in the EV-004 and EV-006 studies. For example, when examining the percentages of patients who achieved a ≥1-point decrease from baseline on the GLS at maximum frown by investigator assessment, 95.9% and 97.4% were responders at days 7 and 30, respectively, following the IT in the EV-004 study; 95.8% and 96.9%, respectively, were responders at these visits following IT in the EV-006 study.^3^ Similarly, 93.9% and 94.1% were responders at days 7 and 30, respectively, following the third RT in the EV-004 study; 96.8% and 96.4%, respectively, were responders at these visits following the third RT in the EV-006 study.^3^

Further evidence of the equivalent potencies of the 2 formulations is shown when comparing the drug exposure and AE profiles of the EV-004 study outcomes reported here with those reported for the EV-006 study. Of note, both formulations contained identical excipients, with a 100-U vial (approximately 4 ng toxin complex) containing 0.5 mg human serum albumin and 0.9 mg NaCl. In both studies, on average, patients qualified for and received 3 treatments. In both studies, among study completers, there was a slight trend towards longer retreatment periods. For example, for those who received 4 treatments in the EV-004 study, the mean sequential intervals between treatments were 94.5, 98.3, and 99.8 days (ranges of 83-128 days, 77-156 days and 79-167 days, respectively); for those who received 3 treatments, the mean sequential intervals were 129.7 and 143.8 days (ranges of 89-238 days and 84-233 days, respectively). Importantly, there was no evidence of shortening retreatment periods that might otherwise have been suggestive of immunogenicity and/or the development of resistance.

In both studies, the percentage of patients who experienced an AE after treatment decreased with repeat exposure. For example, in the EV-004 study, 42.0% of patients experienced 1 or more AEs over the course of this study and 29.5% experienced the event following the IT, representing 70.3% of all patients (104/148) who experienced an AE. Progressively lower percentages of patients experienced AEs following each RT. This trend was also observed for study drug–related AEs and is typical of those reported for RTs of other botulinum toxins utilized for this indication, including onabotulinumtoxinA, abobotulinumtoxinA (Dysport, Medicis Pharmaceutical Corp., Scottsdale, AZ), and incobutlinumtoxinA (Xeomin, Merz Pharmaceuticals GmbH, Frankfurt am Main, Germany); in all studies, the incidence of events was highest after the IT.^6-10^

Few patients (7/352, 2.0%) experienced a serious AE, none of which were study drug related. Few (11/352, 3.1%) experienced an AESI; of these, 5 experienced AESIs assessed as unrelated to study drug. Two AESIs were moderate in severity and all others were mild in severity. None was assessed as severe or serious, and no one withdrew due to an AESI. Six patients (1.7%) experienced a total of 6 AESI assessed as study drug related. Of particular interest and similar to what was observed in the EV-006 study, at 1.4% (1.8% in EV-006), the overall rate of patients with eyelid and/or eyebrow ptosis compares favorably with ptosis rates that have been reported for other toxins in other similarly designed 12- and 13-month-long repeat-dose studies: 23 of 501 onabotulinumtoxinA-treated patients (4.6%)^6^ and 45 of 1200 abobotulinumtoxinA-treated patients (3.8%), respectively.^9^ In our study, 0.9% experienced a related eyelid ptosis event and 0.6% experienced an unrelated eyelid ptosis event. In theory, study drug–related ptosis events such as these could potentially be minimized or eliminated by tailoring the toxin injection sites to accommodate the underlying anatomy of each patient’s target muscles rather than assigning fixed locations 1 cm above the bony orbital rim. Unfortunately, this type of latitude is not afforded in clinical studies designed to achieve FDA approval because it can complicate the regulatory approval process; consequently, neither is it afforded in the instructions for use approved for this type of product and indication.

None of the electrocardiographic findings observed were of concern for the overall cardiac safety of prabotulinumtoxinA. No other findings based on the laboratory hematology, chemistry or urinalysis measures, vital signs, or utilization of concomitant medications were particularly noteworthy.

Although exploratory in nature, evidence of the efficacy of repeat doses of 20 U of prabotulinumtoxinA for the treatment of moderate to severe glabellar lines over the course of 1 year was apparent by all exploratory efficacy measures assessed. As was observed in the parallel repeat-dose EV-006 study,^3^ utilizing each of the GLS at maximum frown, the GAIS, and the SSS, there was a similar pattern of rapid response to treatment in the first week posttreatment (measured at IT day 3 in our study) with peak values observed at the IT day 14 visit and at RT day 7/day 30 visits, as well as no pattern of diminished response with RTs. Similarly, no loss of effectiveness has been observed with RTs of other botulinum toxins approved for this indication.^6-10^ Refer to the publication of the EV-006 results for a discussion of the merits of using a ≥1-point vs a ≥2-point improvement on the GLS to monitor changes to glabellar lines over time as well as the reporting of earlier outcomes within the first week of treatment recorded at IT day 2 (ie, by 48 hours following the IT).^3^

In addition, by study end, 75.9% of patients by investigator assessment who could potentially have experienced a 1-point or greater improvement on the GLS at rest did so; 71.1% did so in the EV-006 study. Improvement in glabellar lines at rest may be a result of further relaxation of hypertonic resting muscles, along with possible soft tissue remodeling as a result of prolonged/long-term muscle relaxation. Further study is warranted to investigate this hypothesis.

Limitations of the EV-004 study parallel those stipulated for the EV-006 study. These include problems inherent to open-label, non-randomized, uncontrolled study design, even when this type of design more accurately reflects normal utilization by the general population. It should also be noted that the directed questionnaire, administered by the investigator or trained study personnel at each visit to ensure the reporting of AEs was comprehensive, was completed in person in a non-anonymous fashion. Of further note, this design element, which was mandated by the FDA, had the potential to lead to the over-reporting of AEs. Reflective of the clinical profile for this type of product, both males and older patients were under-represented. In addition, with 91.8% of study patients identified as White, patients of other ethnicities were also under-represented. Finally, as previously discussed, the formulation of prabotulinumtoxinA utilized in the EV-004 study differed from that of the final commercial formulation.

## CONCLUSIONS

In summary, the safety of RTs of 20 U of prabotulinumtoxinA (formulated utilizing the earlier freeze-drying methodology) for moderate to severe glabellar lines in adult patients was established in this multicenter, open-label, long-term phase II study based on a broad range of outcomes, including AEs and serum antibody testing. Furthermore, an examination of exploratory efficacy outcomes suggests that there is no pattern of diminished effectiveness with RTs.

## Notes

ClinicalTrials.gov Register URL and Identifier: https://clinicaltrials.gov/ct2/show/NCT02184988?term=DWP-450&rank=2
