# Corrigendum to: The First of Two One-Year, Multicenter, Open-Label, Repeat-Dose, Phase II Safety Studies of PrabotulinumtoxinA for the Treatment of Moderate to Severe Glabellar Lines in Adult Patients

**DOI:** 10.1093/asj/sjab229

**Published:** 2021-07-26

**Authors:** Joely Kaufman-Janette, Rui L Avelar, Brian S Biesman, Zoe Diana Draelos, John E Gross, Derek H Jones, Mary P Lupo, Corey S Maas, Joel Schlessinger, Ava Teresa Shamban, Hema Sundaram, Susan H Weinkle, Vernon L Young

In the article “The First of Two One-Year, Multicenter, Open-Label, Repeat-Dose, Phase II Safety Studies of PrabotulinumtoxinA for the Treatment of Moderate to Severe Glabellar Lines in Adult Patients” by Kaufman-Janette et al in *Aesthet Surg J.*, sjaa383, https://doi.org/10.1093/asj/sjaa383, errors were noted in co-author Joel Schlessinger’s name and disclosure statement. A corrigendum is being published to address the following changes that have subsequently been corrected:

In the author list and affiliations, co-author Joel Schlessinger’s full name should read: “Joel Schlessinger,” rather than how it previously appeared.

In the Disclosure section, the following text should read: “Dr Schlessinger is an investor and has served on the advisory board for Evolus; he is also a shareholder of AbbVie, and has served as a researcher for Merz, Galderma, Allergan/AbbVie and Croma Pharma,” rather than how it previously appeared.

